# Optimization of a Mucoadhesive Vaginal Gel Containing Clotrimazole Using a D-Optimal Experimental Design and Multivariate Analysis

**DOI:** 10.3390/polym15092023

**Published:** 2023-04-24

**Authors:** Elena Dinte, Rares Iuliu Iovanov, Andreea Elena Bodoki, Ioana Alina Colosi, Horatiu Alexandru Colosi, Nicoleta Tosa, Oliviu Vostinaru, Ioan Tomuta

**Affiliations:** 1 Department of Pharmaceutical Technology and Biopharmaceutics, Faculty of Pharmacy, Iuliu Hațieganu University of Medicine and Pharmacy, 400012 Cluj-Napoca, Romania; rivanov@umfcluj.ro (R.I.I.);; 2 Department of General and Inorganic Chemistry, Faculty of Pharmacy, Iuliu Hatieganu University of Medicine and Pharmacy, 400010 Cluj-Napoca, Romania; 3 Department of Microbiology, Iuliu Hatieganu, Faculty of Medicine, University of Medicine and Pharmacy, 400349 Cluj-Napoca, Romania; 4 Department of Medical Education, Division of Medical Informatics and Biostatistics, Iuliu Hatieganu University of Medicine and Pharmacy, 400349 Cluj-Napoca, Romania; 5 Molecular and Biomolecular Department, National Institute for Research & Development of Isotopic and Molecular Technologies, 400293 Cluj-Napoca, Romania; 6 Department of Pharmacology, Physiology and Physiopathology, Faculty of Pharmacy, Iuliu Hatieganu University of Medicine and Pharmacy, 400349 Cluj-Napoca, Romania

**Keywords:** polyacrylic acid, polyethene oxides, bioadhesion, vaginal candidiasis, statistical optimization, multivariate analysis, FT-IR

## Abstract

The aim of this study was to develop a suitable clotrimazole (CLT)-loaded mucoadhesive vaginal gel (CLT-MVG) for topical applications in vaginal candidiasis. Ten CLT-MVG formulations were prepared, consisting of mixtures of acid polyacrylic (Carbopol 940) and polyethene oxides, Sentry Polyox WSRN 1105 or 750, according to an experimental D-optimal design, and CLT was suspended at a ratio of 1%. The prepared CLT-MVG formulations were studied in vitro, and the formulation containing Carbopol 940 0.89% combined with PEO 1105 1.39% was identified with the optimal rheological and in vitro bioadhesion properties, ensuring the prolonged release of CLT, with a similarity factor greater than 50, indicating dissolution profile similarity for three batches of the optimized formulation. This optimized formulation showed a pH in the tolerance range, and an adequate ex vivo mucoadhesion time, while the FT-IR studies revealed no interactions between the excipients and CLT. The microscopic analysis identified a mean particle size of suspended CLT of 5.24 ± 0.57 μm. The in vitro antifungal activity of the optimized formulation was tested on twenty strains of *Candida albicans* and proved to be better compared to a marketed clotrimazole preparation, showing a greater inhibition effect (*p* < 0.05). The optimized formulation could be a good candidate for the local treatment of vaginal mycosis.

## 1. Introduction

Vulvovaginal candidiasis (VVC) is the second most common vaginal condition, caused in approximately 90% of cases by Candida albicans species [1]. It is a widespread condition worldwide [2], both in fertile women (during pregnancy) [3] and postmenopause [4], the risk factors being multiple, e.g., the use of antibiotics, diabetes, immunosuppression, and hormonal status, including local vaginal therapy with antimicrobials or hormone replacement therapy [5,6]. Although reports indicate that approximately 70% of women experience at least one instance of VVC throughout their lives, with 50% of women suffering at least one recurrence, detailed epidemiological data are scarce, either because many women with positive Candida cultures are asymptomatic, or because they resort to self-diagnosis and self-medication, the availability of products released without medical prescription being considerable [7]. Besides the specific symptoms, VVC has emotional implications, affects the patient’s quality of life, and also has a significant economic impact [7]. Thus, VVC can cause complications during pregnancy and postmenopause. Although, physiologically, the incidence of postmenopause Candida infection is low, it could increase in the next decade, as a result of the increase in the average age of active women, which, in turn, results in increased expenses for the health systems and a decrease in work productivity [8,9].

The topical application of semi-solid or solid forms in the treatment of VVC is a rational therapeutic approach that allows high local concentrations of the active substance to be obtained, while simultaneously avoiding the adverse reactions associated with the systemic administration of the drug [10,11,12]. Among the commercialized pharmaceutical forms, gels seem to be the products of choice because they are easy to apply and well tolerated, as they alleviate the VVC symptoms through the hydration and lubrication effects conferred by the mucus-like properties of the polymers in their composition [13,14,15,16].

The increased incidence of VVC has stimulated interest in the diversification of the therapeutic strategies aimed at ensuring the eradication of Candida infections by using formulations with good tolerance and optimized therapeutic efficacy [17,18]. In this context, bioadhesive semi-solid formulations represent advantageous alternatives as they ensure the prolonged retention of the active substance, good distribution at the level of the vaginal tract, and the efficient interaction of the active principle with the targeted pathogens. The development of a vaginal product represents a great challenge, because the formulation must overcome the limitations imposed by the physiological particularities (gravity, vaginal secretions) while ensuring improved pharmacokinetic characteristics and an adequate safety and efficacy profile of the active ingredient [19,20,21,22].

Bioadhesive polymers frequently used in the formulation of vaginal systems are natural, e.g., hyaluronic acid, sodium alginates, starch, chitosan, or natural gums, or synthetic compounds, e.g., polyacrylic acid derivatives, cellulose derivatives (hydroxyethylcellulose, hydroxypropylcellulose, hydroxypropylmethylcellulose), and polyethylene oxides. In order to obtain characteristics that comply with the therapeutic objective, mixtures of different polymers are used, with the aim of obtaining gels with a viscosity that allows a comfortable application, good tolerance, increased adhesiveness, and the prolonged release of the drug [23,24,25,26,27]. Clotrimazole (CLT) is an imidazole derivative widely used for the treatment of mycotic infections of the genitourinary tract. It is very effective locally and has no major side effects [28]. Including CLT in a polymeric matrix that combines balanced spreading, viscosity, bioadhesion, and prolonged release characteristics could ensure improved antimycotic efficiency.

The use of response surface methodologies in the design of experiments applied in the formulation and the optimization of pharmaceutical dosage forms is a useful tool that allows the screening of appropriate excipients and the simultaneous evaluation of the effects of formulation variables on the characteristics of interest of the product so that its clinical performance be maximized [29].

The objective of the study was the development of a vaginal mucoadhesive gel with CLT, having optimized viscosity and bioadhesion properties, showing good tolerance and the prolonged release of CLT at the level of the vaginal mucosa, in order to be successfully used in the local treatment of vaginal infections with Candida.

## 2. Materials and Methods

### 2.1. Materials

Polyethylene oxides (PEO Sentry Polyox WSRN 1105 NF (PEO_1105_) and WSRN 750 NF (PEO_750_) were a generous gift from Colorcon Limited (Kent, UK). Polyacrylic acid, Carbopol 940 (C_940_, B.F. Goodrich Chemical Co., Cleveland, OH, USA), glycerol, and mucin from porcine stomach, Type III, were from Sigma-Aldrich (Darmstadt, Germany). Triethanolamine and polyethylene glycol 400 (PEG 400) were from Merck (Darmstadt, Germany). CLT was provided by Beijing Double Crane, Pharmaceutical Co., Ltd. (Beijing, China). All other reagents were of analytical grade.

### 2.2. Design of Experiments (DoE) and Preparation of CLT-MVG Formulations

Ten formulations of CLT-loaded mucoadhesive vaginal gel (CLT-MVG) were prepared by combining the hydrophilic polymers in different ratios, according to a D-optimal experimental design, with three factors and two levels. The independent variables were the component fractions and the type of hydrophilic polymer used in the preparation of the gels, polyethylene oxides (PEOs) and Carbopol 940 (C_940_) (Table 1). D-optimal designs are known as model-specific designs that solve design constraints and require minimum experimental runs. The dependent variables were the rheological, the in vitro adhesion parameters, and the in vitro CLT release characteristics (Table 1).

PEOs and C_940_ were dispersed in deionized water, separately. In the case of C_940_, glycerol was used as a humectant (1%), and the aqueous dispersion was neutralized with triethanolamine. The bioadhesive matrix was prepared by mixing the appropriate amounts of the polymeric aqueous dispersions so that the final CLT-MVG formulations presented the composition provided in the experimental plan. CLT was incorporated in a concentration of 1% by precipitation from solution in PEG 400 (1:8, m/m, dissolved at 50 °C), which was added gradually, by stirring at 75 rpm with a mechanical stirrer. The preparations were kept at room temperature for 24 h and were subsequently subjected to additional studies.

### 2.3. Determination of the Pharmaceutical Characteristics of CLT-MVG Formulations Used as DoE Responses

#### 2.3.1. Determination of Spreading Capacity

The in vitro spreadability was studied by measuring the spreading surface (cm^2^) occupied by 1 g of the CLT-MVG formulation placed between two 20 × 20 cm glass plates [30]. The mass of the upper plate was standardized at 125 g, and a 500 g mass was placed on the device. The spreadability was determined after 1 min and the results represent the average of three determinations (*n* = 3).

#### 2.3.2. Determination of Detachment Force

**Figure 1:**
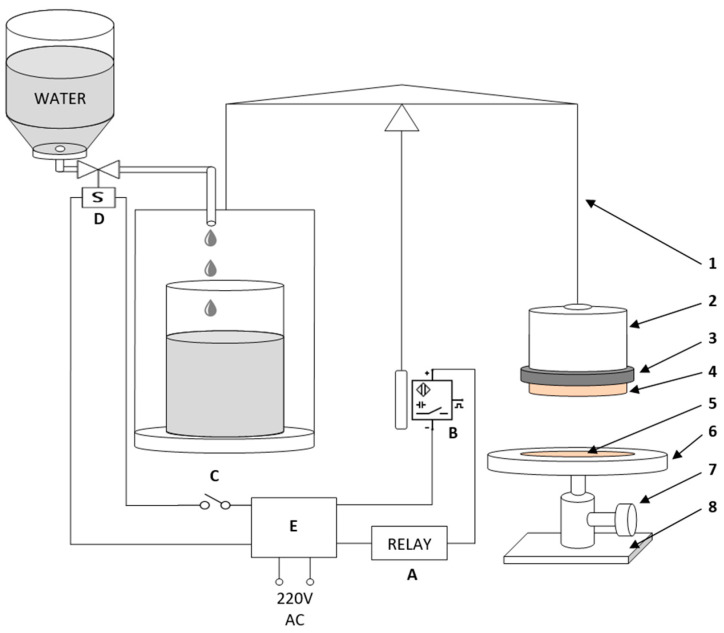
The in vitro bioadhesion testing experimental device. Bioadhesion scale: 1. detachable arm; 2. metallic cylinder; 3. membrane attachment ring; 4. semi-permeable membrane; 5. product compartment containing the bioadhesive preparation; 6. product support; 7. adjustment/fastening screw; 8. fixed support, electrical circuit: A. relay; B. proximity sensor; C. relay control; D. electro-valve (12V D.C.); E. electrical transformer (12V DC).

The in vitro bioadhesion capacity of the CLT-MVG formulations was determined by measuring the detachment force of a membrane brought into contact with the studied sample. An experimental device (Figure 1) was set up using a modified two-armed balance (Mechaniki precyzyincj Zaklady, Poland), adapted according to the literature [31]. The studies were carried out at 25 ± 1 °C using a synthetic membrane (polymeric cellulose film), immersed in a 1% aqueous mucin dispersion. The left-side platter of the scale was used to counterbalance the scale’s right side and to determine the mass needed to detach the membrane from the formulation (the water collected in a beaker). The right-side platter was replaced with a metal cylinder suspended by a detachable arm. The membrane was fastened on the cylinder using an attachment ring. The product support containing the CLT-MVG formulation was placed under the metallic cylinder, and the relative position of this compartment was adjusted with a screw, until the equilibrium of the scale was reached, and then this position was maintained by fastening the adjustment screw. In this position, the needle points at the “0” position of the scale and it is in front of a proximity sensor. In this configuration, the electro-valve is open, and the water can flow freely into the beaker. The compartment has a cylindrical cavity (the contact surface equals to 133 mm^2^) in which the sample is placed using a spatula. Before the beginning of the adhesion test, the sample from the cylindrical cavity and the hydrated membrane stayed in contact for 60 s. After the initial contact time, the water was allowed to flow, with a flow rate of 60 drops/minute, and collected in the beaker on the left side of the balance. When the needle changes position (the membrane detaches from the tested sample), the electro-valve closes, because the proximity sensor is triggered, and water flow is interrupted. Thus, the necessary mass to detach the membrane from the sample can be determined, and the detachment force was calculated according to the formula

Detachment force (mN) = maximum weight needed for detachment (g) × gravitational acceleration (m·s^−2^). (1)

#### 2.3.3. Determination of Thixotropy Index, Yield Stress, Apparent Viscosity, and Consistency Index

The viscosity measurements were performed on a Brookfield Model DV III Ultra Viscosimeter (Brookfield Engineering Laboratories, Inc., Middleboro, MA, USA), using a spiral adaptor.

The determinations were made at 25 °C ± 0.5, with three replicates for each experiment. The rheograms were obtained by measuring the shear stress for different shear rates (in the range 0.3–100 s^−1^); the flowing type was analyzed using the Herschel–Burkley rheological model (Equation (2)):

(2) $$\tau =\tau_{0}+\;K\cdot \gamma^{n}$$

where

*τ* = shear stress (Pa);
*γ* = shear rate (s^−1^), *n* is the non-Newtonian index (0 < *n* < 1);
*K* (consistency index) is a factor related to the apparent viscosity of the gel.

The term *τ_0_* is the yield stress and represents the shear stress necessary for the flow to begin.

The rheological parameters such as yield stress, apparent viscosity (at 20 s^−1^), consistency index, and thixotropy index were calculated using the Rheocalc var.3.0 Program.

#### 2.3.4. In Vitro Drug Release and Kinetic Release Evaluation

In vitro release studies of CLT were performed on all gel formulations using the USP 24 Method (Dissolution Apparatus II, Pharma Test PT-DT7, 0.1 M citrate–phosphate buffer pH 5.5, 1% Tween 80, 37 ± 0.5 °C). First, 1–2 g of CLT-MVG formulation was placed in a dialysis bag (Spectra/Por Cellulose Ester Membrane MWCO: 5000–8000 Da) immersed in the dissolution medium, stirred at a 10 rpm rate. Five-milliliter samples were collected periodically and replaced with fresh dissolution medium. The CLT concentration in the medium was determined using an HPLC-UV validated method (Agillent, 1100, Santa Clara, CA, USA) at 261 nm. The analytical column was a Zorbax SC C18 (5 μm, 4.6 × 150 mm) and the mobile phase consisted of acetonitrile and 0.1% phosphoric acid (45:55, *v*/*v*; *t_R_* = 2 min). The exact amounts of CLT released at each time interval were calculated using a calibration curve (r^2^ = 0.9996).

In order to confirm the similarity of the dissolution profiles for the optimized formulation, the similarity factor was calculated for three replicates. The similarity factor, *f*_2_ [32], directly compares the similarity between the percentage of drug dissolved per unit time for a test and a reference product. The similarity factor (*f*_2_) is a logarithmic transformation of a sum-squared error of differences between the test (*T_j_*) and the reference product (*R_j_*) over all time points:

(3) $$f_{2}=50\log\left\{{\left[1+\left(\frac{1}{N}\right)\overset{n}{\underset{j=1}{\sum }}{\left|T_{j}-R_{j}\right|}^{2}\right]}^{-0.5}\right\}\times 100$$

To evaluate the mechanism of kinetic release of CLT from the dosage form, correlation coefficients (R^2^) and release rate constants (K) for various models (Korsmeyer–Peppas, Higuchi, first-order, and zero-order model) were determined for all studied CLT-MVG formulations [33]. The mathematical models were fitted to individual dissolution data with the regression module of SigmaPlot 8.

### 2.4. Optimization Procedure and Multivariate Analysis

The experimental data obtained in the studies performed on all ten formulations generated by the experimental design were fitted to a second-order polynomial model, allowing the prediction of the effect of the formulation variables on the rheologic characteristics, bioadhesive capacity, and drug release profile using a small number of experiments. In this mathematical approach, each experimental response Y can be represented by a square equation of the response surface:

Y = b_0_ + b_1_X_1_ + b_2_X_2_ + b_3_X_3_ + b_11_X_1_^2^ + b_22_X_2_^2^ + b_33_X_3_^2^ + b_12_X_1_X_2_ + b_13_X_1_X_3_ + b_23_X_2_X_3_ (4)

where X_1_, X_2_, X_3_ are the independent variables, and the coefficients are as follows:

b_i_ represents the estimation of the main effects of the factors X_ijk_;
b_ii_ represents the estimation of the second-order effects, and
b_ij_ and b_ijk_ are the estimations of the interactions between X_i_ and X_j_.

The equation allows the study of the effects of each formulation factor and their interactions on the considered response. The experimental data were fitted to the chosen mathematical model using the Modde for Windows computer program ver.13.0 (Sartorius Stedim Data Analytics AB, Sweden). In addition, a multivariate analysis method (SIMCA P13, Sartorius Stedim Data Analytics AB, Sweden) was used to evaluate the influence of the rheological and adhesive parameters on CLT’s in vitro release kinetics.

These procedures allowed us to calculate the optimal area in the studied experimental range for the rheological and adhesive characteristics considered of interest for the designed CLT-MVG formulation.

### 2.5. Additional Characterization of the Optimized CLT-MVG Formulation

#### 2.5.1. FT-ATR-IR Spectroscopy

The FT-ATR-IR spectra were recorded using a Jasco FT-IR 4100 spectrometer equipped with a ZnSe crystal for the direct acquisition of the attenuated total reflection (ATR) infrared spectra of solid samples. The spectra were recorded in the range of 4000–550 cm^−1^ with a spectral resolution of 1 cm^−1^, using a thermal stabilized TGS detector.

#### 2.5.2. CLT Particle Size

The optimized CLT-MVG formulation was analyzed in terms of the characteristics of the suspended CLT particles. The microscopic images were obtained using an advanced set-up with an optical inverted Olympus IX71 microscope, and the CLT particle size distribution was determined using the CellSense Software ver. 1.3, 2010 (Olympus Corporation, Tokyo, Japan) for image recording and analysis.

#### 2.5.3. pH Determination

The pH of the optimized CLT-MVG formulation was determined using a calibrated Mettler Toledo MP 225 digital pH meter. Here, 1 g of sample was dispersed in 100 mL deionized water and the electrode was allowed to reach equilibrium. The results represent the average of three determinations (*n* = 3) [34].

#### 2.5.4. Ex Vivo Mucoadhesion Time

The time required for the detachment of the optimized CLT-MVG formulation from a natural membrane was determined according to a protocol described in the literature [35,36,37], using an adapted tablet disintegrator (Erveka GmbH, Langen, Germany). Freshly isolated porcine vaginal mucosa, obtained from a slaughterhouse, was stored at 2–8 ± 0.5 °C in sterile isotonic saline and used within 48 h. Then, 6 × 4 cm mucosa pieces were cut and fixed with cyanoacrylates on double-sided adhesive tape previously fixed on a Plexiglas plate. Next, 1 mL gel was applied to the mucosa with a syringe and spread using a spatula so that the gel occupied the surface of a 4 × 2.5 cm rectangle. After 10 min of contact, the plate was fixed in the device in a vertical position and the test proceeded by raising and lowering the plate in citrate–phosphate buffer solution, pH 5.5, at 37 ± 1 °C, until the complete elution of the gel. The average of five determinations was calculated.

#### 2.5.5. In Vitro Antifungal Activity Evaluation

The antifungal activity of the optimized CLT-MVG formulation was tested on 20 strains of *Candida albicans*: 2 ATCC (American Type Culture Collection) strains, *Candida albicans* (ATCC^®^ 10231™) and *Candida albicans* (ATCC^®^ 90028™), and 18 strains of *Candida albicans* from vaginal secretions isolated by three clinical laboratories in Cluj-Napoca, Romania.

Culture medium and testing method: A Mueller–Hinton medium supplemented with 2% glucose (to ensure adequate growth of yeasts) and 0.5 mg/mL methylene blue (providing better definition of the inhibition zone diameters) was used. The method for antifungal disk diffusion susceptibility testing of yeasts was used according to the approved CLSI M60-Ed1 guideline [38].

Several well-isolated colonies from a 24 to 48 h pure culture on Sabouraud dextrose agar were homogenized in 0.85% NaCl to obtain turbidity equivalent to the 0.5 McFarland standard (corresponding to a suspension of 1–5 × 10^6^ CFU, Colony Formation Units). Sterile swabs were soaked in the inoculum suspension and used to inoculate the Mueller–Hinton agar plates by streaking the entire surface. After drying for 10–15 min, six-millimeter-diameter wells were cut from the agar using a sterile cork borer. Predetermined volumes of 50 μg for each tested compound were poured into the individual wells as follows: 50 μg/well of the tested CLT gel, 50 μg/well of a commercial CLT cream, 50 μL/well of CLT solution, and 50 μg/well of saline control solution, respectively. The plates were incubated at 35 °C. Zone diameters (mm) of significant growth reduction (80%, after 24–48 h) were measured and approximated to the nearest whole millimeter [38].

Data analysis: The inhibition zone diameters of the optimized CLT-MVG formulation were compared to those of 1% clotrimazole solution, clotrimazole cream, and saline solution, respectively, using the Student’s paired *t*-test in Microsoft Excel. The threshold for statistical significance was set at α = 0.05. In the absence of a gel with CLT on the pharmaceutical market, the in vitro antifungal activity of the optimized CLT-MVG formulation was compared to a product of equal potency, Canesten^®^ 1% cream (10 mg/g) (positive control).

## 3. Results

### 3.1. Design of Experiments (DoE) Analysis

The results obtained for the dependent variables (denoted as Y) in the performed studies are presented in Table 2.

The spreading surface is an essential characteristic of semi-solid preparations, and for the studied CLT-MVG formulations, it was found in the range of 15.55 ± 1.21–26 ± 0.78 cm^2^, considered adequate to allow easy spreading during application, a factor that increases patient compliance and ensures optimal contact between the formulation and the mucosa [39]. The rheological recordings showed that all the studied CLT-MVG formulations exhibited non-Newtonian pseudoplastic behavior, with yield stress and thixotropic behavior (measured thixotropy index greater than 5 [40], i.e., obtained values in the range 8.82 ± 0.76–11.8 ± 1.25). Thixotropic behavior is desirable during the preparation and manipulation of the gel as it allows a loss in viscosity when the shear stress increases and the recovery of the viscosity after it decreases [40]. The values obtained for yield stress, a measure of the initial resistance of the formulation to flow, were within 45.13 ± 0.28–391.5 ± 10 Pa, and the viscosity measured at 20 s^−1^ varied in the range of 2412.9 ± 98–7691.7 ± 79 Pa·s, values suitable for vaginal application. The bioadhesive capacity, evaluated by studying the in vitro detachment force and with values between 90 ± 2.12 and 250 ± 13.68 mN, is comparable to that previously reported [35] and within the acceptable range to ensure an optimal retention time after application on the vaginal mucosa.

To determine the levels of variables that ensure optimal adhesivity, viscosity, and drug release characteristics, mathematical relationships were generated between the dependent and independent variables. The model was fitted to the data with the software described earlier (Table 3). The initial model was refined to include only those terms for which the significance level was below *p* < 0.05. In terms of the quality of data fitting, the model was adequate in the case of the responses Y1–Y10 accounting for more than 90% of the responses’ variation. The reproducibility of the model was over 90% for Y2–Y5, Y7, Y9. The prediction capacity of the model was over 80% for Y3, Y4, Y9, with a mean reproducibility in the case of the Y2 and Y5 responses and acceptable for Y1 and Y6, Y7.

**Figure 2:**
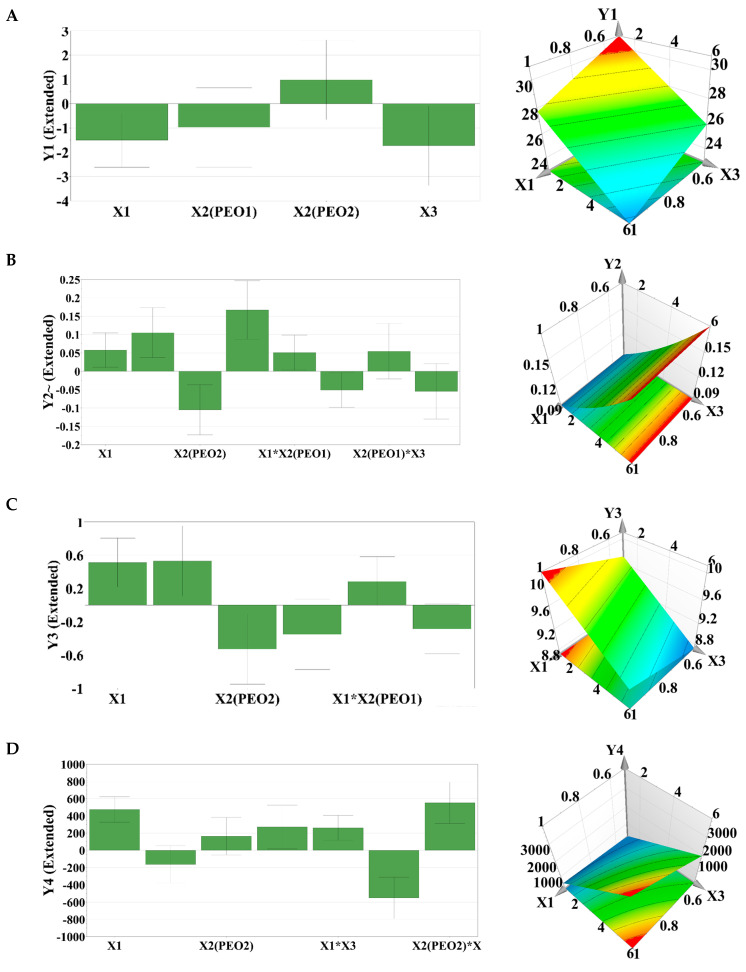

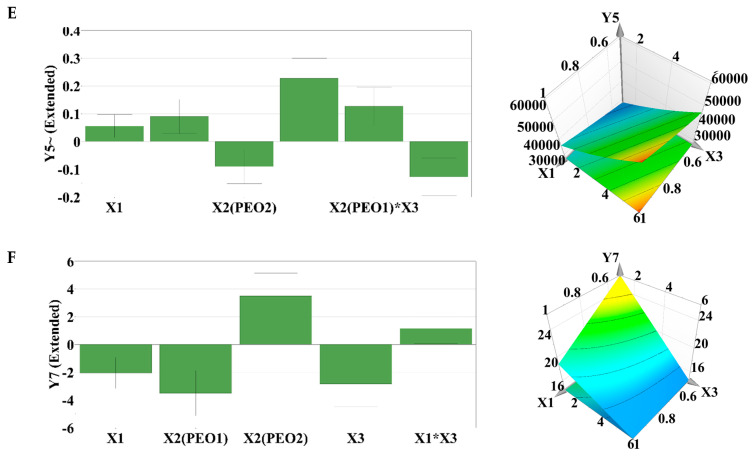
Centered and scaled coefficients (**left**) and response surface plot (**right**) illustrating the influence of C_940_ and PEOs on responses (Y1-Y5 and Y7). (**A**): Y1 -spreading capacity; (**B**): Y2-detachment force; (**C**): Y3-thixotropy index; (**D**): Y4-yield stress; (**E**): Y5-viscosity at 20 s^−1^; (**F**): Y7-K–Peppas.

The quantitative effect of the formulation factors on the studied experimental responses was illustrated by response surfaces and contour plots. The response surface methodology is a collection of mathematical and statistical techniques used to model and analyze problems, where a response of interest is influenced by multiple variables and the objective is to optimize this response. Regarding the evolution of the studied rheological parameters, a decrease in the spreading surface (Y1) was identified by increasing the concentrations of polymers in the formulation. PEO_1105_ had a negative influence on the response, while PEO_750_ had a positive influence. These results can be explained by the increased consistency of the formulations based on C_940_ and PEO_1105_, which reduced the spreading capacity (Figure 2A).

The detachment force (Y2) increased with the increase in the concentrations of polymers in the formulation, PEO_1105_ having an obvious influence. In addition, increasing the concentration of PEO_750_ led to a decrease in the detachment force, and this behavior is in agreement with the viscosity results obtained in the case of the studied formulations (Figure 2B).

The thixotropy index (Y3) increased with increasing amounts of C_940_ and PEO_1105_ in the formulation, while PEO_750_ had a negative influence. An interaction effect between C_940_ and PEO_1105_ was observed with a negative influence on the response, which caused a reduction in the thixotropy index (Figure 2C). The yield stress (Y4) increased with the increase in the concentration of C_940_ in the formulation (Figure 2D), and the viscosity increased with the increase in the concentrations of polymers in the preparation (Figure 2E), this effect being stronger in the case of PEOs. Interaction phenomena between PEO_1105_ (X2) and the concentration of PEO_1105_ (X3) were also observed, which determined the increase in viscosity, as well as interaction phenomena between PEO_750_ and the concentration of PEO_750_, which led to a decrease in viscosity. No quadratic effects on responses were observed. Figure 2F highlights the influence of the formulation factors on the kinetic constant of the Peppas equation, which showed the best fitting of the release data (R^2^ > 0.99), this constant being a measure of the release rate of CLT from the studied CLT-MVG formulations. Increasing the concentration of C_940_ and PEO_1105_ determined a decrease in the release rate, while an increase in the concentration of PEO_750_ in the formulation determined an increase in the release rate.

### 3.2. In Vitro Clotrimazole Release from Mucoadhesive Vaginal Gels

**Figure 3:**
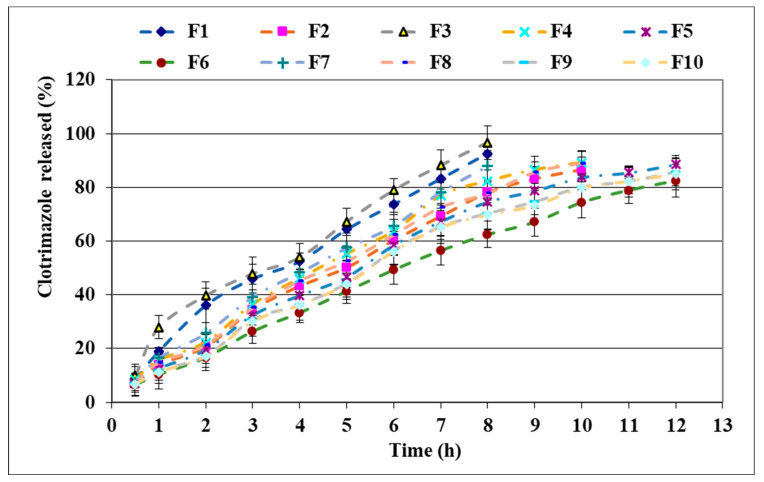
In vitro release of CLT from mucoadhesive vaginal gels (F1–F10, according to Table 1).

Analysis of the release profiles of CLT (Figure 3) revealed that all the studied gels showed prolonged release of the drug. The fastest release was observed in the case of the formulations containing PEO_750_ and the lowest C_940_ concentration. Thus, the amount of CLT released within 8 h was greater than 80% in the case of three formulations, the extent of the release and the release rate decreasing in the order F3 > F1 > F7. The release rate is higher in the case of the gel containing 0.5% C_940_ and 3% PEO_750_ (F3), followed by the gel containing 0.5% C_940_ and 1% PEO_1105_ (F1), and by F7 containing a higher ratio of PEO_750_ (6%). When a higher concentration of C_940_ (1%) was associated with the same concentrations of PEO_750_ and PEO_1105_ (formulations F2, F4, F8), the release varied in the same manner (F4 > F8 > F2), but the release process was prolonged for a total duration of 10 h. The F2 formulation containing 1% C_940_ associated with 1% PEO_1105_ released the drug slower as compared to the formulation in which the C_940_ was associated in the same concentration (1%) with 3% PEO_750_ (F4). The formulations in which C_940_ was associated in concentrations of 0.75% or 1% with PEO_1105_ in concentrations greater than 1% (F5, F6, F9, F10) ensured the prolonged release of CLT, over a duration of 12 h.

Table 4 provides a summary of the model fitting and statistical parameters for the release kinetics of the studied gel formulations. The drug release kinetics may be best described by the Peppas model, followed by the zero-order model. The Peppas model generated n-values in the range of 0.6946–0.8536, indicating that the release of CLT follows a non-Fickian behavior, in which the diffusion phenomena from the three-dimensional network structure of the gel are over-imposed on the swelling ones. The zero-order release behavior may suggest that the release of CLT was controlled by the erosion constant of the matrix [33].

### 3.3. Multivariate Analysis

**Figure 4:**
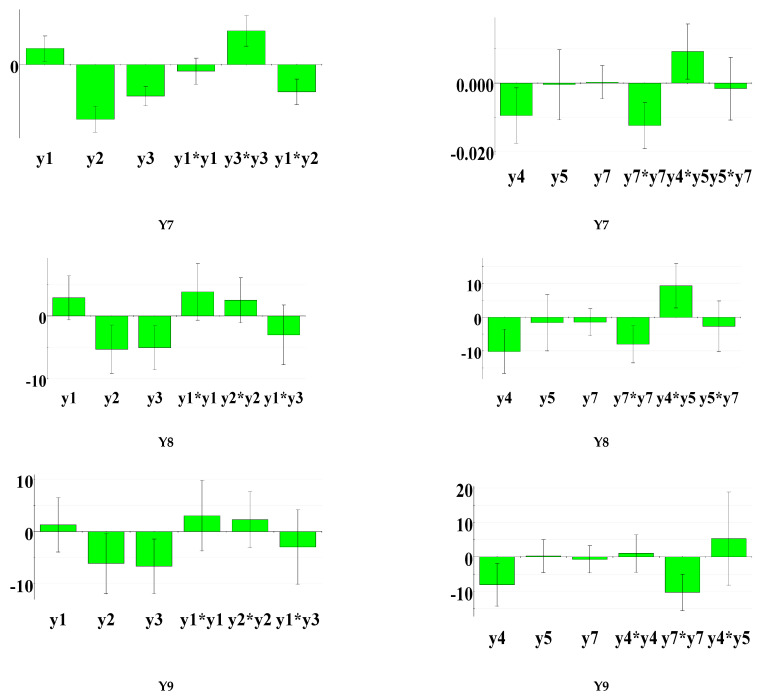

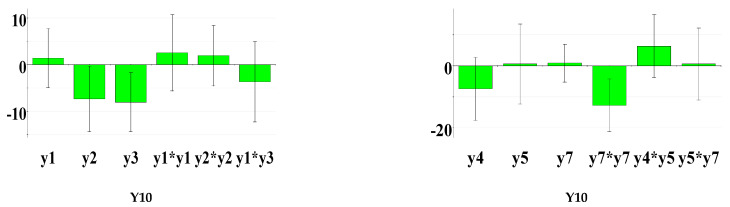
Multivariate analysis Y1, Y2, Y3 versus Y7, Y8, Y9, Y10 (**left**) and Y4, Y5, Y6 versus Y7, Y8, Y9, Y10 (**right**). Y1-spreading capacity; Y2-detachment force; Y3-thixotropy index; Y4-yield stress; Y5-viscosity at 20 s^−1^; Y6-consistency index; Y7-K–Peppas; Y8-CLT released at 2 h; Y9-CLT released at 5 h; Y10-CLT released at 8 h.

In order to perform a more detailed analysis of the factors that influenced the characteristics of interest of the designed product, a multivariate analysis was applied, which allows the identification of correlations between different data sets. Thus, the influence of responses Y1–Y6 (rheological parameters, in vitro adhesion strength) on the release speed and the amount of CLT released, at 2, 5, and 8 h (Y7–Y10), was analyzed. The analysis of the centered and scaled coefficients shows that an increase in the spreading capacity determined the increase in the release rate and the released amount of CLT in the first 2 h, but the release was not significantly influenced at 5 and 8 h of the study. Increasing the thixotropy index caused a decrease in the amount of CLT released and in the release rate (Figure 4, left). In vitro adhesion strength is a parameter that varies depending on the nature and the concentration of the bioadhesive polymers, sometimes being proportional to the rheological parameters, and, in this case, it negatively influenced the CLT release parameters.

Surprisingly, the increase in viscosity did not significantly influence the drug release from all prepared gels (Figure 4, right). The yield stress reduced the rate of release and the amount released only in the first 2 h of the study, but viscosity–yield stress interaction effects were observed, which resulted in an increase in release at the beginning of the study. The consistency index did not have a significant influence on the release parameters. The results obtained can be explained by the influence of the polymers in the formulation, which can increase the viscosity, a fact that influences the magnitude and release rate of CLT. Increasing the ratio of C_940_, PEO_1105_, and PEO_750_ in the formulation resulted in increased viscosity, but an important aspect is the behavior of the gel in contact with the aqueous fluids, where the nature of the polymers has a major impact. An increase in the PEO_750_ ratio can induce an increase in the viscosity and consistency, but an increase in the release rate was also noted as a result of the fact that this polymer forms a lax gel, easily dispersible in water.

### 3.4. Experimental Design Validation and Similarity of the Dissolution Profiles

Analyzing both the influence of the formulation factors and of some responses on the characteristics of the studied CLT-MVG formulations, constraints were applied to the chosen mathematical model so that the designed product would present a good spreading surface (allowing the application of the product on the vaginal mucosa), optimal viscosity and increased adhesive strength (so that the product remains for as long as possible at the place of application), and medium thixotropy (which allows the preparation of a homogeneous product and the maintenance of physical stability during storage), but would also ensure the prolonged release of the CLT for a period of 12 h. The targeted values for the optimized formulation selected were as follows: spreading surface: 22–26 cm^2^; detachment force: 120—200 mN; thixotropy: 10.0—11.4; yield stress: 100–200 Pa; viscosity at 20 s^−1^: 3000–4000 Pa·.s. The parameters that were not significantly influenced by the formulation factors were not included. The isoresponse curves were superimposed and the optimization procedure generated the optimal formulation, which was calculated for the following values: Carbopol_940_ 0.89% and PEO_1105_ 1.39%. This formulation was prepared in triplicate (A, B, and C) and studied for rheological, in vitro adhesive, and drug release characteristics. The observed responses (Y1–Y5) were close to the theoretically estimated ones (Table 5).

Under the studied experimental conditions, the optimized formulation showed the prolonged release of CLT for 12 h, and the values obtained for f2 were greater than 50 (between 50 and 100), which shows the similarity of the dissolution profiles. The f2 values calculated in this study for A versus B, B versus C, and A versus C were 90.22, 96.41, and 93.84, respectively. These findings suggest that the investigated in vitro drug release profiles were therefore similar.

### 3.5. Additional Characterization of the Optimized Formulation

#### 3.5.1. FT-ATR-IR Studies

**Figure 5:**
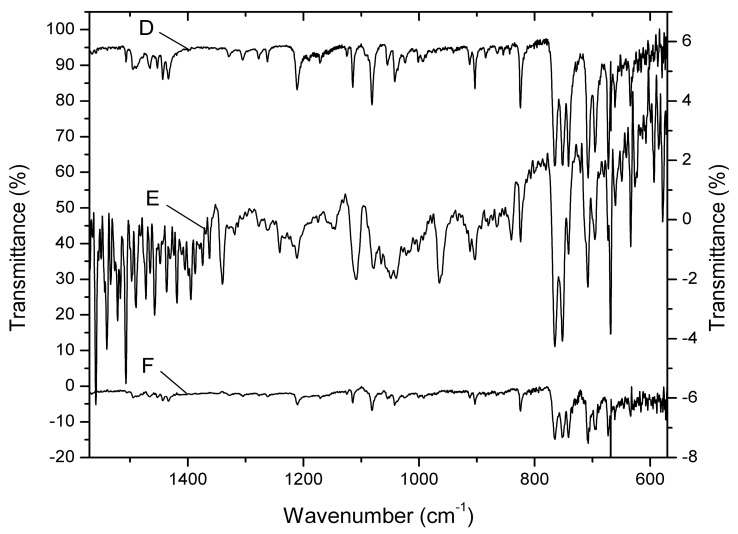
FT-ATR-IR spectra of the optimized CLT-MVG formulation. D-pure CLT; E-residual spectrum of CLT after the subtraction of placebo from the optimized formulation’s spectra; F-residual spectrum of CLT after the subtraction of the spectrum of 1:1 (*w*/*w*) PEO_1105_:C_940_ excipient mixture from the spectrum of 1:1:1 (*w*/*w*) PEO_1105_:C_940_:CLT mixture.

These studies investigated the compatibility of CLT with the constituents of the polymeric matrix. Any possible interaction, physical or chemical, between the excipients and CLT, leading to vibrational shifts in the local chemical bonds, can be identified by IR spectroscopy [41]. Any major difference between the spectra of pure CLT and the one of the active compound incorporated in the polymeric matrix is proof of such interactions that could possibly compromise the long-term stability of the optimized CLT-MVG formulation. Therefore, the vacuum-dried optimized formulation and the placebo FT-ATR-IR spectra were also recorded. After the subtraction of the placebo’s spectrum from the optimized formulation’s, the resulting residual spectrum shows the characteristic vibration bands of pure CLT (Figure 5D,E). The strong out-of-plane C-H bending vibrations of the mono- and di-substituted aromatic rings (phenyl and o-chlorophenyl) of CLT are clearly visible in the range of 765–695 cm^−1^. The weak aromatic C-H stretching bands (3100–3000 cm^−1^) are barely visible in the case of pure CLT and they are missing in the residual spectra, most probably due to the low concentration of the antifungal agent in the mucoadhesive formulation.

A clearer image of any possible interactions between the used polymers and CLT was investigated by preparing a 1:1:1 (*w*/*w*) mixture of the two excipients (PEO_1105_ and C_940_) and CLT, the active compound. The spectrum of the 1:1 mixture of the two excipients was subtracted from the recorded FT-ATR-IR spectrum (Figure 5F).

Once again, the residual spectra were compared with the reference spectrum of CLT, without any noticeable changes or shifts in the characteristic bands. The results demonstrate that the structure of CLT remains unaltered when embedded in the mucoadhesive vaginal gel’s polymeric matrix.

#### 3.5.2. CLT Particle Size, pH, and Ex Vivo Mucoadhesion Time

**Figure 6:**
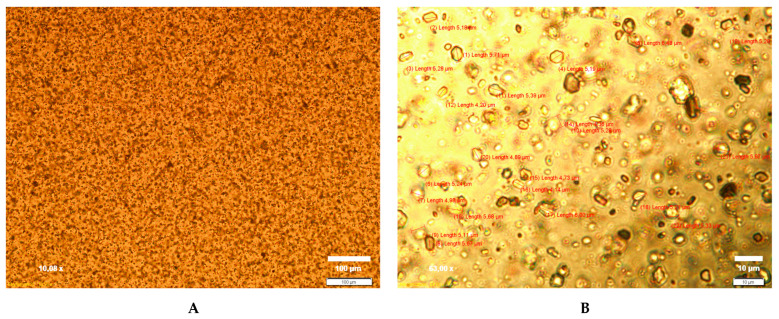
CLT particles suspended in the optimized CLT-MVG formulation. (**A**) Magnification of 10.08× (scale 100 μm); (**B**) magnification of 63× (scale 10 μm).

The suspended CLT particles in the optimized CLT-MVG formulation were between 4.14 and 6.48 μm in size, with a mean particle size of 5.24 ± 0.57 μm, as shown in Figure 6.

The pH is an important parameter to be considered in the formulation [42], and it was 5.5 ± 0.81 for the optimized formulation. Considering the need to neutralize the aqueous dispersion of C_940_ and to achieve a pH between 5 and 10 to ensure gelation, the results can be considered acceptable to ensure tolerance within the vaginal mucosa during temporary use in the treatment of a mycotic infection.

The average time required for the complete removal of the optimized CLT-MVG formulation from porcine vaginal mucosa was 71.20 ± 8.02 min, considered acceptable to ensure optimal retention after vaginal application.

#### 3.5.3. In Vitro Antifungal Activity of the Optimized CLT-MVG Formulation

**Figure 7:**
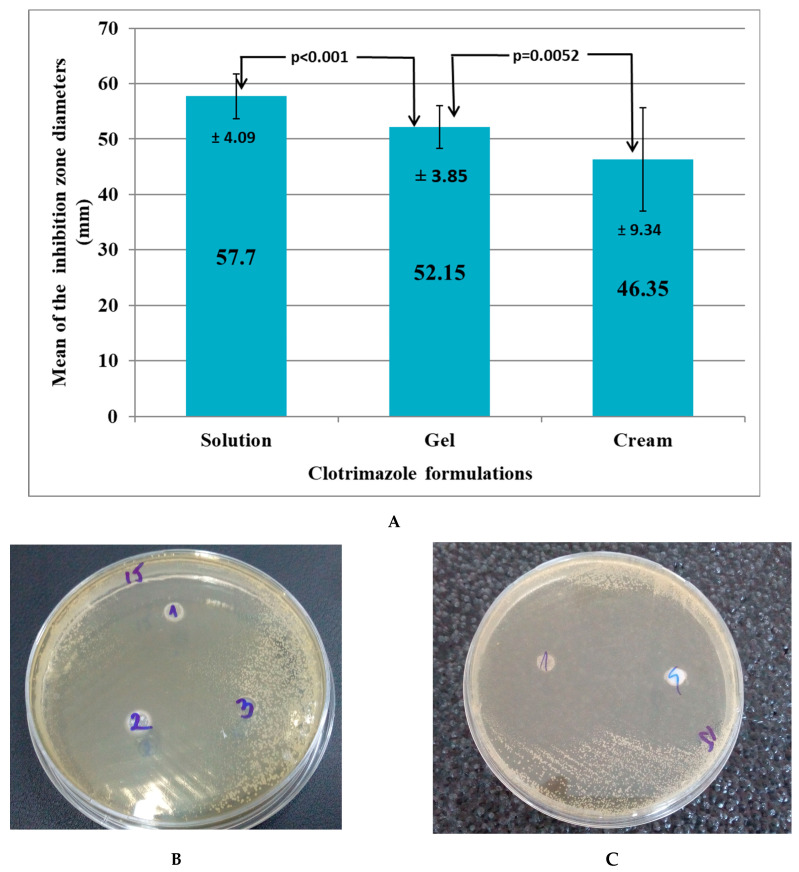
In vitro antifungal activity of the optimized CLT-MVG formulation. (**A**) Mean inhibition zone diameter of the evaluated optimized CLT-MVG formulation, compared to a clotrimazole solution and to a marketed clotrimazole cream (average ± SD); (**B**) diameter of inhibition zone for optimized CLT-MVG formulation (1), clotrimazole solution 1% (2), and sterile saline control (3); (**C**) diameter of inhibition zone for optimized CLT-MVG formulation (1) and marketed clotrimazole cream (Canesten^®^) (4).

The mean inhibition zone diameter of the optimized CLT-MVG formulation was 52.15 mm, with a minimum diameter of 47 mm and a maximum diameter of 61 mm. The mean inhibition zone diameter of the CLT solution was 57.7 mm (Figure 7A). Therefore, the mean difference compared to the CLT gel increased by 5.55 mm for the CLT solution (*p* < 0.001—Student’s paired *t*-test). The mean inhibition zone diameter of the marketed CLT cream was 46.35 mm. This translated into an increase in the mean difference of 5.8 mm in favor of the optimized CLT-MVG formulation: *p* = 0.0052—Student’s paired *t*-test (Figure 7B,C). All saline inhibition zone diameters were absent.

## 4. Discussion

In this study, CLT-MVG formulations were prepared based on mixtures, in different proportions, of C_940_ and two types of polyethylene oxides, PEO_1105_ and PEO_750_, according to a D-optimal experimental design. In all formulations, CLT was incorporated by suspension, at a concentration of 1%. The obtained gel formulations were homogeneous, with the opaque appearance characteristic of suspensions, and odorless.

The rheological studies highlighted appropriate characteristics regarding viscosity, thixotropy, initial flow resistance, and spreadability, in agreement with similar reports [42] and correlated with the specific requirements for vaginal applications. The rheological parameters of vaginal formulations are of fundamental importance since the shear rate of the formulation is high during preparation and vaginal application. Minor changes in composition can produce essential changes in viscosity, thixotropy, or yield stress, parameters that should be maintained at optimal values for proper handling and preparation. Usually, optimal viscosity values increase the retention time and adhesion properties of the formulation; reduced viscosity favors the removal of the product from the site of administration, while increased values are correlated with difficulties in handling and with local irritation effects.

The amount of CLT released, as well as the release rate, are influenced by the drug’s solubility in the release medium and by the physicochemical characteristics of the formulation (viscosity, type of polymer matrix) [43]. The higher the gel viscosity is, the lower the rate of drug release. Thus, since the formulations containing C_940_ and PEO_1105_ have higher viscosity as compared to those based on C_940_ associated with PEO_750_, the release rate of CLT from the former decreases. Regarding the mechanism of CLT release from the studied CLT-MVG formulations, it can be best described by the Peppas model, with n values in the range of 0.69–0.85, indicating a combined effect of formulation factors. Swelling phenomena of the matrix are associated with CLT diffusion from the viscous gel. The increase in the amount of CLT released, as well as the rate of CLT released in the case of formulations based on PEO_750_, is explained by the faster disintegration of the formulation in water, since PEO_750_ has a lower degree of polymerization and forms gels with lower viscosity. These results are also reflected by the higher “n” values, indicating zero-order release and suggesting that the release of CLT is controlled by the erosion of the gel matrix [33]. The average size observed for the suspended CLT particles was 5.24 ± 0.57 μm, which allows the homogeneous dispersion of CLT in the gel matrix, optimal dissolution in vaginal secretions, and a lack of irritation phenomena. Furthermore, the optimized CLT-MVG formulation exhibited a pH value that ensures vaginal tolerance and a good ex vivo mucoadhesion time. The FT-IR studies did not detect any interaction phenomena between CLT and excipients.

The evaluation of in vitro antifungal activity, performed for both the control *Candida albicans* strains and the *Candida albicans* strains isolated from vaginal secretions, revealed quite large inhibition zone diameters (mean diameter = 52.15 mm) in the presence of the optimized CLT-MVG formulation. Even though the difference between the mean inhibition zone diameter for the optimized CLT-MVG formulation and the mean inhibition zone diameter for the CLT solution was highly significant (*p* < 0.001—Student’s paired *t*-test), a mean difference of only 5.55 mm is not relevant in practice, as long as all diameters are situated above 45 mm (min diameter = 47 mm, max diameter = 61 mm). The difference between the mean inhibition zone diameter for the optimized CLT-MVG formulation and that for the Canesten^®^ cream was also statistically significant (*p* = 0.0052—Student’s paired *t*-test), the optimized CLT-MVG formulation proving to have better in vitro diffusion as compared to the commercial CLT preparation. The evaluated optimized CLT-MVG formulation proved to possess excellent in vitro diffusion properties, being intermediate between those of an CLT cream formulation and those of a CLT solution.

## 5. Conclusions

CLT-MVG formulations were prepared according to a D-optimal experimental design, allowing us to obtain a formulation with optimized properties in terms of viscosity, spreading, and bioadhesion, complying with the standards required for vaginal application. The in vitro studies highlighted the prolonged release of CLT, in agreement with the Peppas model, *n* > 0.5, and demonstrated encouraging results for the anti-Candida activity. The formulation optimized in this study offers an alternative strategy for local treatment in VVC.

## Figures and Tables

**Table 1:** The experimental design (independent variables, dependent variables, and matrix).

Independent Variables (Formulation Factors of DoE)						
No.	Name		Symbol	Levels		
				–1	0	+1
1	Carbopol 940 ratio		X_1_	0.5	0.75	1
2	PEO type		X_2_	PEO_1105_		PEO_750_
3	PEO ratio	PEO_1105_	X_3_	1	2	3
		PEO_750_		3	4.5	6
The dependent variables (Response of DoE and characteristics of the mucoadhesive vaginal gel)						
No.	Name		Symbol			
1	Spreading capacity (cm^2^)		Y1			
2	Detachment force (mN)		Y2			
3	Thixotropy index		Y3			
4	Yield stress (Pa)		Y4			
5	Viscosity at 20 s^−1^ (Pa.s)		Y5			
6	Consistency index (Pa.s)		Y6			
7	K–Peppas (h^−1^)		Y7			
8	Clotrimazole released at 2 h (%)		Y8			
9	Clotrimazole released at 5 h (%)		Y9			
10	Clotrimazole released at 8 h (%)		Y10			
The matrix experimental design						
Exp. Name	X_1_	X_2_	X_3_			
F1	0.5	PEO_1105_	1			
F2	1	PEO_1105_	1			
F3	0.5	PEO_750_	3			
F4	1	PEO_750_	3			
F5	0.5	PEO_1105_	3			
F6	1	PEO_1105_	3			
F7	0.5	PEO_750_	6			
F8	1	PEO_750_	6			
F9	0.75	PEO_1105_	2			
F10	0.75	PEO_1105_	2			

**Table 2:** Matrix of CLT-MVG responses (denoted as Y1–Y10).

Exp. Name	Spreadability (cm^2^)	Detachment Force (mN)	Thixotropy Index	Yield Stress (Pa)	Viscosity at 20 s^−1^ (Pa·s)	Consistency Index (Pa·s)	K_KP_ Peppas (h^−1^)	CLT Released at 2 h (%)	CLT Released at 5 h (%)	CLT Released at 8 h (%)
	Y1	Y2	Y3	Y4	Y5	Y6	Y7	Y8	Y9	Y10
F1	25.50 ± 1.86	90 ± 2.12	10.20 ± 1.21	100.70 ± 22	2412.9 ± 98	10796 ± 201	19.441 ± 11	36.11 ± 2.31	64.33 ± 3.10	92.55 ± 8.11
F2	18.85 ± 0.98	120 ± 10.40	11.40 ± 2.34	155.30 ± 17	2684.7 ± 23	8099 ± 332	13.621 ± 22	20.55 ± 1.67	50.11 ± 2.22	78.16 ± 7.45
F3	26.00 ± 0.78	110 ± 12.01	9.21 ± 0.98	89.20 ± 12	3162.3 ± 45	17664 ± 203	22.540 ± 18	39.74 ± 1.34	67.25 ± 3.01	96.70 ± 9.01
F4	21.22 ± 0.97	125 ± 11.02	9.77 ± 1.13	169.10 ± 13	4290.5 ± 56	19019 ± 123	15.568 ± 77	22.32 ± 0.98	55.32 ± 0.76	82.33 ± 5.08
F5	20.41 ± 0.87	140 ± 12.83	9.94 ± 0.88	45.13 ± 0.28	4471.7 ± 25	35576 ± 350	14.231 ± 58	19.43 ± 0.65	46.52 ± 1.08	74.55 ± 4.56
F6	17.71 ± 1.03	250 ± 13.68	11.80 ± 1.25	145.10 ± 11	7691.7 ± 79	28649 ± 284	10.814 ± 87	16.54 ± 0.78	41.28 ± 1.12	62.41 ± 3.44
F7	22.00 ± 1.11	170 ± 2.27	8.82 ± 0.76	200.80 ± 14	4924.7 ± 88	14846 ± 180	14.725 ± 91	25.66 ± 1.38	57.48 ± 0.77	87.88 ± 6.86
F8	15.55 ± 1.21	180 ± 9.89	8.88 ± 1.03	391.50 ± 10	5921.1 ± 96	8923 ± 210	14.235 ± 67	21.32 ± 1.11	52.44 ± 1.04	77.71 ± 8.02
F9	25.05 ± 1.35	160 ± 11	10.40 ± 1.83	128 ± 5.41	3788.2 ± 55	34645 ± 198	13.173 ± 39	17.22 ± 1.09	44.28 ± 1.78	70.22 ± 6.76
F10	25.10 ± 1.92	159 ± 3.89	10.80 ± 1.23	120 ± 6.22	3756.2 ± 48	34520 ± 205	12.926 ± 45	17.06 ± 1.33	43.99 ± 3.01	69.77 ± 4.92

**Table 3:** Summary of fit.

Response	R^2^	Q^2^	*p*-Value	*p*-Error	F-Value	Model Validity	Reproducibility
Y1	0.79	0.47	0.018	0.448	7,64	0.80	0.86
Y2	0.97	0.69	0.002	0.439	34.39	0.79	0.97
Y3	0.95	0.83	0.010	0.525	15.91	0.84	0.92
Y4	0.99	0.88	<0.001	0.500	63.87	0.83	0.98
Y5	0.93	0.60	0.005	0.521	15.44	0.84	0.91
Y6	0.83	0.45	0.029	0.170	2.85	0.37	0.99
Y7	0.90	0.48	0.011	0.381	10.74	0.76	0.94
Y8	0.87	0.70	0.046	0.355	20.95	0.60	0.97
Y9	0.99	0.85	0.004	0.443	53.99	0.55	0.97
Y10	0.87	0.63	0.020	0.453	8.29	0.80	0.89

R^2^—fraction of the variation in the response explained by the model; Q^2^—predictive power of the model; *p*-values; *p*-error; F-value—the ratio of the mean regression; model validity—the extent to which the measurement corresponds to real-life situations; reproducibility—ability to produce the same output if the input is the same; Y_1_—spreading capacity; Y_2_—detachment force; Y_3_—thixotropy index; Y_4_—yield stress; Y_5_—viscosity at 20 s^−1^; Y_6_—consistency index; Y_7_—K–Peppas; Y_8_—CLT released at 2 h; Y_9_—CLT released at 5 h; Y10—CLT released at 8 h.

**Table 4:** Kinetic models of CLT release from studied CLT-MVG formulations (F1–F10, according to Table 1).

Kinetic Models	Parameter	Formulation									
		F1	F2	F3	F4	F5	F6	F7	F8	F9	F10
Korsmeyer–Peppas model	K_KP_	19.441	13.621	22.40	15.568	14.231	10.814	14.725	14.235	13.173	12.926
	*n*	0.7474	0.8219	0.6946	0.7832	0.7616	0.8304	0.8536	0.8128	0.7769	0.7831
	R^2^	0.9976	0.9974	0.9933	0.9949	0.9930	0.9984	0.9988	0.9976	0.9923	0.9926
Higuchi model	K_H_	29.223	24.894	31.023	26.426	24.289	21.301	26.791	25.848	23.418	23.065
	R^2^	0.9626	0.9485	0.9689	0.9535	0.9562	0.9485	0.9454	0.9536	0.9558	0.9516
First-order model	K_1_	0.2214	0.1612	0.2479	0.1796	0.1523	0.1207	0.1885	0.1720	0.1415	0.1388
	R^2^	0.9869	0.9851	0.9769	0.9838	0.9873	0.9896	0.9840	0.9860	0.9883	0.9880
Zero-order model	K_0_	12.384	9.5326	13.083	10.086	8.4698	7.4706	11.2679	9.7311	8.0722	8.0588
	R^2^	0.9748	0.9879	0.9558	0.9794	0.9733	0.9900	0.9935	0.9882	0.9774	0.9771

Here, R^2^ represents the correlation coefficient. The K_KP_, K_H_, K_1_, K_0_ are rate constants following the Korsmeyer–Peppas, Higuchi, first-order, and zero-order mathematical models, respectively. The *n* is the release exponent, which explains the release mechanism.

**Table 5:** Optimized formulation.

Composition	Responses			
	Name	Predicted Values	Obtained Values	Bias (%)
X1: C_940_—0.89%	Y1 (Spreading surface)	26.1183 cm^2^	23.22 ± 4.22 cm^2^	−11.10
X2: PEO_1105_	Y2 (Detachment force)	141.72 mN	150.91 ± 8.92 mN	6.48
X3: PEO_1105_—1.39%	Y3 (Thixotropy index)	11.29	10.72 ± 2.19	−5.05
	Y4 (Yield stress)	133.83 Pa	142.87 ± 7.18 Pa	6.75
	Y5 (Viscosity at 20 s^−1^)	3170 Pa·s	3834 ± 109 Pa·s	20.95

## Data Availability

Not applicable.
